# Button battery ingestion in children is potentially fatal: assessing and raising awareness of a decades old problem

**DOI:** 10.1017/S0022215125103782

**Published:** 2026-02

**Authors:** Anmol Mahesh, Ivan Keogh, Khalid Majeed

**Affiliations:** 1 Department of Otolaryngology Head and Neck Surgery, University Hospital Galway, Ireland; 2 Academic Department of Otorhinolaryngology, University of Galway, Ireland

**Keywords:** emergency ORL, caustic ingestion, public health, dysphagia, swallowing, oesophagus

## Abstract

**Background:**

Button batteries are a common household item that are, unfortunately, attractive to young children. If ingested, they are corrosive and potentially fatal. Button battery ingestion is frequently unwitnessed, delaying the diagnosis. In the USA, approximately 6,000 accidental ingestions occur annually (2.2 deaths per year over a decade on average). Community awareness of this danger appears to be low.

**Methods:**

We conducted a 22-question online questionnaire-based study to assess and raise awareness of this exceptional childhood risk.

**Results:**

A total of 561 survey responses were analysed; 77 per cent were female, and 60 per cent were aged 30–50. Despite 87 per cent using button batteries, 65 per cent did not consider their safety, and 68 per cent found existing packaging warnings inadequate. Notably, 80 per cent recognised the potential for fatality, but 88 per cent were unaware that a spoonful of honey could delay this corrosive process.

**Conclusion:**

Challenges persist regarding the design and marketing of button batteries and public awareness of their ingestion. Action is required to prevent further tragedies.

## Introduction

Foreign body ingestion is a common occurrence in young children. The peak incidence of foreign body ingestion occurs in children aged six months to six years old.^1^

Research has demonstrated that more than 80 per cent of ingested foreign bodies will pass spontaneously without issue.^2^^,^^3^ Button battery ingestion comprises a small percentage of all foreign body ingestions. However, the unique morbidity and mortality associated with button battery ingestion makes them particularly noteworthy amongst other ingested foreign bodies.^4^ Button batteries are ubiquitous in the environment and are used to power many common household products including toys, watches, calculators, flashlights and remote controls.^5^ The button battery market is immense, valued at $4.9 billion in 2024.^6^

**Figure 1.**
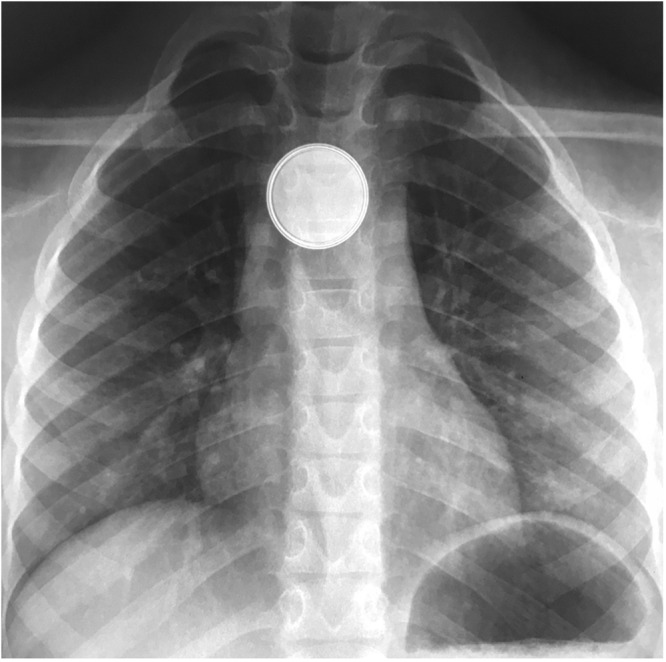
Halo sign (double ring sign) on anterior–posterior view chest x-ray.^20^

**Figure 2.**
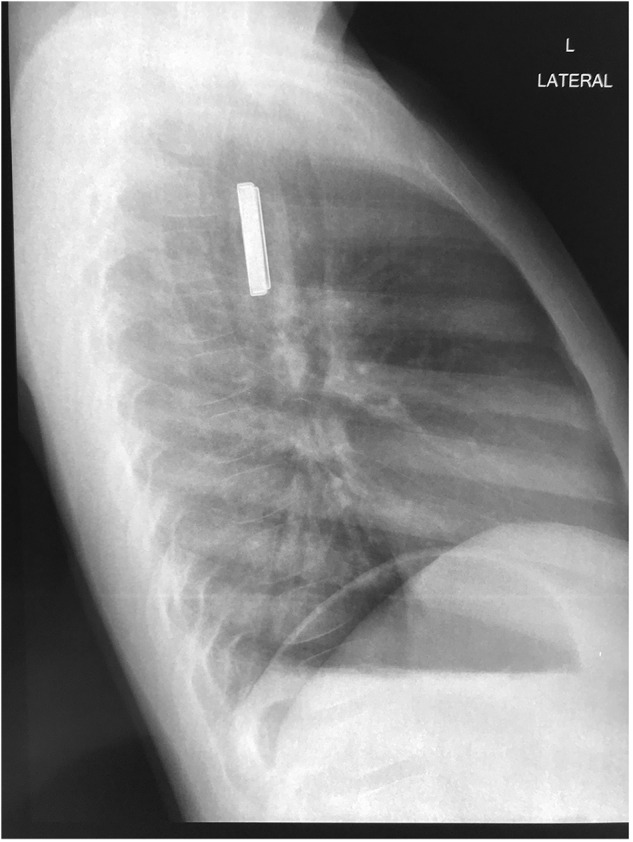
Step-off sign on lateral view chest x-ray.^20^

Button batteries present a unique hazard to the upper aerodigestive tract. There are multiple mechanisms of injury at play, the most detrimental being the generation of an electric current in the proximity of the oesophageal mucosa, resulting in the electrolysis of water molecules.^4^^,^^7^ This mechanism creates an alkaline hydroxide solution at the negative pole of the battery that is caustic to the mucosa, resulting in progressive liquefactive necrosis of the oesophagus and adjacent tissues.^4^^,^^7^ Complications of this process include oesophageal perforation, pneumothorax, pneumomediastinum, oesophageal stenosis, aortoesophageal fistula, massive haemorrhage, tracheoesophageal fistula and death.^4^^,^^8^^,^^9^ Although mucosal damage can occur within 2 hours, these complications may take days to weeks to manifest.^9^ Button battery ingestion in children is a time-sensitive situation because the damage to the mucosa manifests within minutes.^4^ The immediate safe removal of an impacted button battery is mandated. A hallmark of the diagnosis of button battery ingestion is the appearance of the “halo” sign (Figure 1) on anterior–posterior x-ray view and the “step-off” sign (Figure 2) on lateral x-ray view.^4^^,^^8^

An increasing number of button battery ingestions have been seen worldwide as they become more prevalent in consumer electronics.^4^ According to the National Poison Data System in the USA, button battery ingestions have increased from 745 in 1985 to 3467 cases in 2019.^10^

**Figure 3.**
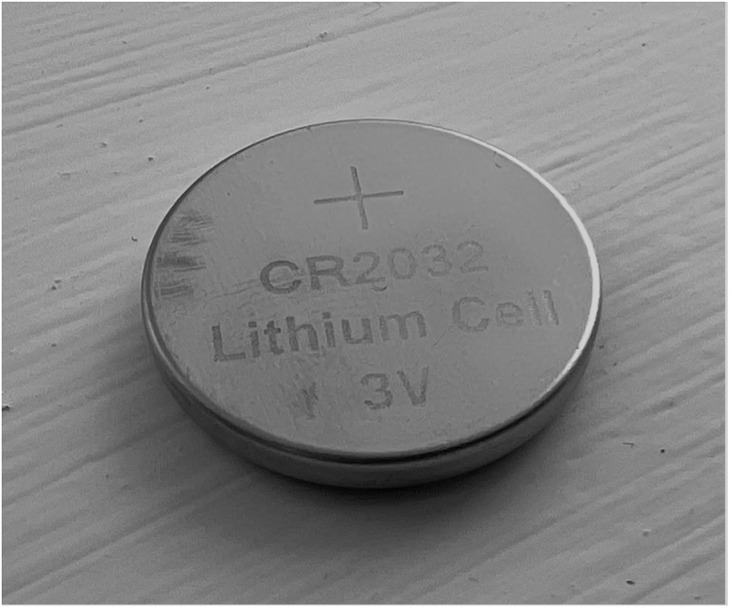
Positive pole of a CR2032 button battery (20-mm diameter).

**Figure 4.**
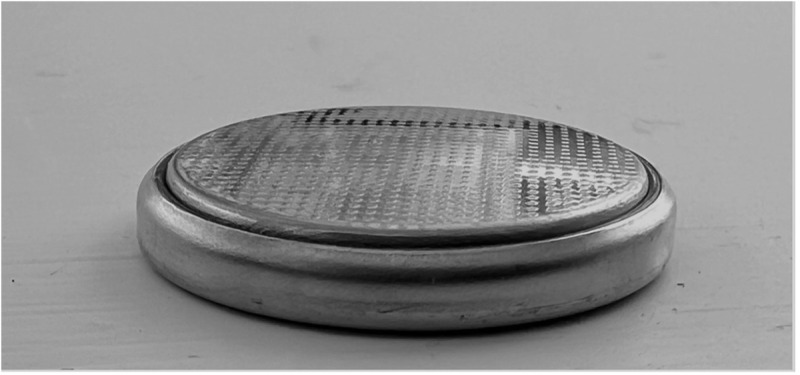
Lateral view of a CR2032 button battery depicting its “stepped” appearance (3.2-mm height).

In addition to this, patient outcomes have worsened in recent years as there are increased numbers of large diameter (greater than or equal to 20 mm) and higher voltage button battery ingestions.^4^ CR2032 button batteries are the foremost culprit of such ingestions. CR2032 batteries are 3V lithium ion round batteries with a diameter of 20 mm (Figure 3) and a height of 3.2 mm (Figure 4).

Batteries sized at greater than or equal to 20 mm are associated with more than 90 per cent of fatalities as they are more likely to lodge in a child’s narrow upper oesophagus.^11^ An analysis of 8648 cases reported to National Battery Ingestion Hotline in the USA found that the batteries were obtained directly from electronics in 62 per cent of cases or were from loose or discarded button batteries in 30 per cent of cases.^5^ Of the cases of large (greater than or equal to 20 mm) button battery ingestion, 37 per cent were batteries intended for remote controls.^5^ Thus, parental education plays a huge role in mitigating the occurrence of button battery ingestions, with the increasing numbers of ingestions possibly suggesting that awareness is low. Much of the currently published research puts focus on the pathophysiology and emergency hospital-care protocols rather than health education. There also appears to be a lack of national data on the subject in Ireland as there is no dedicated national hotline for battery ingestion.

Hospital protocols surrounding the treatment of button battery ingestion have been created that generally aim for the removal of an impacted button battery within 2 hours followed by irrigation of the site with an acidic solution.^4^^,^^8^^,^^9^ The latter half of this management strategy has expanded to become part of the general advice for parents who suspect that their child has ingested a button battery. A study performed by Anfang *et al*. found honey to be an effective means of protecting against pH-mediated oesophageal damage, owing to its acidity that counteracts the alkalinity created at the negative pole of the battery and its viscosity that allows the honey to coat the battery, thereby acting as a physical barrier.^12^ Carers are advised to administer a spoon of honey to their child if they suspect button battery ingestion.

We hypothesise that awareness of the mechanisms at play, the complications and the mitigation strategies surrounding button battery ingestion is low. Our research aims to gather data on the awareness among the general population of the hazards of button battery ingestion.

## Materials and methods

This cross-sectional online study was performed in August 2023. Participants were required to be over the age of 18.

Ethical approval was obtained from the Galway Clinical Research Ethics Committee to perform the study (Approval number C.A. 3005).

A self-completed survey using Google Forms was used to collect responses from the participants. The survey comprised two sections. The first contained 25 questions designed to assess the participants knowledge regarding paediatric button battery ingestion. The second section collected the participants demographic information.

The survey was disseminated by a combination of physical posters containing a QR code distributed in the Galway City area and digital dissemination of the webpage link to individuals, institutions and workplaces. A total of 561 responses were collected.

Data were analysed using IBM SPSS Statistics for Windows, Version 28. Data were expressed as frequency and percentage.

## Results and analysis

### Demographics of the participants

A total of 561 responses were recorded; 114 participants (20.3 per cent) were age 20-30; 183 participants (32.6 per cent) were in the 30–40 years age band, with a further 152 (27.1 per cent) aged 40–50 years; 429 (76.5 per cent) participants were female, and 126 (22.5 per cent) were male. A total of 527 (93.9 per cent) report Ireland as their primary place of residence; 408 (72.7 per cent) participants reported being or having been a caregiver to a child under the age of 6. A total of 223 (39.8 per cent) participants were healthcare professionals. Demographic data of the participants is detailed in Tables 1 and 2.

**Table 1.** Age of participants

Age of participants (years)	Frequency	Percentage
<20	18	3.2
20−30	114	20.3
30−40	183	32.6
40−50	152	27.1
50−60	73	13.0
60−70	17	3.0
>70	4	0.7

**Table 2.** Gender of participants

Gender of participants	Frequency	Percentage
Female	429	76.5
Male	126	22.5
Other	4	0.7
Prefer not to say	2	0.4

### Awareness of the participants of paediatric button battery ingestion

Despite 487 (87 per cent) of participants using button batteries, only 202 (36 per cent) considered button battery safety when buying toys for children. Meanwhile, 384 participants (68 per cent) found existing packaging warnings for items containing button batteries inadequate in informing them of the potential risks to children. Of note, 301 participants (53.7 per cent) did not know or were unsure of how to create a safe home environment with regards to button batteries, and 446 participants (79.5 per cent) did not feel they could recognise the symptoms of button battery ingestion in a child, were it to occur. Encouragingly, 508 participants (90.6 per cent) were aware that a depleted button battery can still be corrosive, and 450 participants (80.2 per cent) recognised the potential for fatality, but 465 (83 per cent) were unaware of the negative pole’s responsibility for the corrosion. A total of 92.3 per cent of participants were aware that children under the age of four years are the most common age group implicated in cases of button battery ingestion, but 290 participants (51.7 per cent) did not know that the process of alkalinisation and liquefactive necrosis begins almost immediately, and 89 per cent were unaware that a spoonful of honey could delay this process. Participants generally had a low awareness of the specific complications of button battery ingestion. 252 participants (44.9 per cent) were aware of secondary oesophageal scarring and only 214 (38.1 per cent) were aware of haemorrhage as specific complications.

Amongst those aged 20 to 30, the age group most likely to have a child(ren) under the age of five, 28.1 per cent considered button battery safety when buying toys. Only 16.7 per cent believed manufacturer warnings on button battery-containing items adequately informed them of the potential danger button batteries pose to children; 79 per cent were not aware of the symptoms of button battery ingestion; and only 14.9 per cent were aware that honey could be used to delay the corrosive damage caused by button battery ingestion.

## Discussion

Button battery ingestion is an area of research that is receiving more attention. The 2009–2018 period has seen a 187 per cent increase in medical button battery publications when compared to previous years, with a rise in non-US publications in particular.^13^ Despite this, 71 per cent of articles were case reports and case series, and the remainder focus on clinical detection and treatment algorithms.^13^ Little attention has been placed on the public health aspect of this issue.

Predictably, our research has confirmed the ubiquity of button batteries in the daily lives of the population. A large majority (87 per cent) of participants reported using them in their everyday lives. However, button battery safety was not a major concern for many of the participants. A total of 47 per cent of the participants stated that they did not consider safety, with a further 17.5 per cent answering “uncertain.”

Our research has shown that there is a general awareness of the danger of paediatric button battery ingestion. A total of 93 per cent of the participants were aware that urgent medical attention is warranted for cases of button battery ingestion. However, the awareness of the unique dangers posed by button batteries is low. For example, 43 per cent were unaware that the process of liquefactive necrosis begins immediately upon impaction of the button battery in the upper oesophagus.

A total of 68 per cent of participants found the warnings on the packaging of items containing button batteries inadequate in informing consumers about the potential risk button batteries pose to young children. It is outside of the scope of this paper to discuss the various European Union (EU) regulations, legislation and directives that mandate particular safety warnings on products, particularly toys, that contain button batteries but suffice it to say, the participants of our study found the existing mandatory safety warnings to be inadequate.

Frequently, paediatric button battery ingestion is unwitnessed by the child’s carer. The presenting symptoms of button battery ingestion are non-specific and can include vomiting, drooling, dysphagia, odynophagia, irritability, coughing, stridor and shortness of breath.^8^ A total of 53 per cent of participants were unaware of these symptoms. This is worrying as a delay in diagnosis increases the risk of complications.^11^

Awareness of these complications was relatively low amongst participants. Although major complications and deaths are uncommon each year (death occurred in 1.6 per cent of button battery ingestions in USA in 2019), the severity of such outcomes warrant efforts to increase public awareness.^10^ Although Ireland’s Health Service Executive (HSE) has a website for poisoning in young children, we suggest that a dedicated button battery ingestion/aspiration website be created.^14^

In recent years, there has been some positive progress around button battery safety. On the 16 August 2022, President Biden signed “Reese’s Law” in the United States of America, which mandates federal safety requirements for button, cell and coin batteries.^15^ The aim of this is to reduce the risk of button battery ingestion in children. As a consequence of Reese’s Law, new regulations by the Consumer Product Safety Commission require that button battery compartments on consumer products necessitate the use of a tool or two independent and simultaneous hand movements to open.^16^

Participants were generally unaware of simple home remedies which may slow mucosal damage after button battery ingestion. The ingestion of electrically insulating materials such as honey (which is additionally protective due to its acidic nature) or sucralfate can delay the electrically mediated necrotic process that occurs and can buy valuable time until hospital-based interventions can be accessed.^12^ Amongst the participants, awareness of this was surprisingly low. Only 11.4 per cent had knowledge of this potentially life-saving intervention.

Alternative agents such as olive oil have been suggested by the literature but have proven to be inferior.^17^ Safe removal via endoscopy requires general anaesthesia as well as senior anaesthetic and surgical staff.

Of note, only 20 per cent of the participants of our study were aged 20 to 30. Knowledge and awareness of button battery safety is most pertinent for parents in this age group. We found that awareness and lack of awareness amongst this group was similar to that of the overall group of participants. It would be apt for future public-health–focused studies in this area to ensure that a substantial proportion of the studied participants exist within this age group.

The rise of short-form social media may present a novel means of tackling public awareness of paediatric button battery ingestion. TikTok parenting advice videos are gaining popularity, with hundreds of videos existing that educate parents on the use of honey for button battery ingestion. The “shock-factor” of said videos has resulted in many of them receiving hundreds of thousands of “likes.”

Unique alternative measures for increasing button battery safety in the home have been suggested in the literature. For example, the taping of button batteries intended for disposal by parents has been suggested and has proven effective in studies.^18^ Alterations to button battery design have also been suggested, such as pressure-activated button battery coatings that are only active inside the housing of the device the battery is powering.^19^ Such a design could prove to be particularly effective as 61.8 per cent of button batteries ingested by children were attained directly from items that utilise the batteries.^5^ However, such measures would require radical and costly shifts in the manufacturing process. Button battery manufacturers need to proactively engage and take responsibility for button battery safety.

Fortunately, there have been some positive steps taken by manufacturers recently. Energizer® have implemented a “3-in-1 Child Shield^TM^” technology for their retailed button batteries that includes secure packaging requiring scissors to open, a bitter tasting coating on the battery and a saliva-activated blue dye coating on the battery.

Finally on another positive note, the first Global Button Battery Task Force Meeting took place on the 17 July 2024. Hosted by Professor Ian Jacobs from the Children’s Hospital of Philadelphia, they hope to address the ongoing concerns about the dangers of button batteries when accidentally swallowed by children.

- Paediatric button battery ingestion can result in significant morbidity and mortality
- There are few studies assessing public awareness of this fact
- In this study, the awareness of the respondents of the significant dangers associated with paediatric button-battery ingestion is low
- Despite 87 per cent of respondents using button batteries, 65 per cent did not consider their safety an issue
- A total of 68 per cent found existing safety warnings on the packaging of items containing button batteries inadequate
- A total of 83 per cent were unaware that the negative pole is responsible for the corrosive process
- A total of 88 per cent did not know a spoonful of honey could delay this corrosive process

## Conclusions

Button battery ingestion is a time-sensitive paediatric emergency with the potential for significant morbidity and mortality. This study has shown that awareness of the specific dangers associated with button battery ingestion and potentially life-saving interventions, such as the ingestion of honey, is inadequate amongst the general public. This study highlighted the need for improving public awareness, the burden of which is potentially lessened by the far-reaching influence of social media.

The study also found that, whilst there exist safety warnings on items containing button batteries, members of the public still feel that these are inadequate in fully informing them of the potential risks to children. Regulation changes with regards to the required format of these warnings may be warranted in service of improving public health education and consumer safety.

Ultimately, significant challenges remain regarding the design, marketing and public awareness of button batteries.
